# The impact of gender, age and hearing loss on tinnitus severity

**DOI:** 10.1590/S1808-86942010000100004

**Published:** 2015-10-17

**Authors:** Patricia Ciminelli Linhares Pinto, Tanit Ganz Sanchez, Shiro Tomita

**Affiliations:** 1 Otorhinolaryngologist/master's degree student, coordinator of the tinnitus outpatient unit of the Rio de Janeiro Federal University (UFRJ); 2 Professor of otorhinolaryngology of the Medical School, Sao Paulo University (USP), national coordinator of the Tinnitus Support Group. Coordinator of the research group in tinnitus of the Clinical Hospital, Sao Paulo University (USP); 3 Full professor of otorhinolaryngology of the Medical School, UFRJ. Head of the otorhinolaryngology unit of the Clementino Fraga Filho University Hospital, UFRJ

**Keywords:** hearing loss, gender, age, tinnitus

## Abstract

Tinnitus is a symptom present in approximately 15% of the world population. Most patients are between 40 and 80 years of age; the prevalence above 60 reaches 33%. About 20% have moderate to severe impact in the quality of life but the factors associated with the tinnitus annoyance are not completely known.

**Aim:**

The objective of this study is to evaluate the relationship between age, gender and hearing loss on tinnitus annoyance.

**Materials and methods:**

68 patients were evaluated at the tinnitus center at our hospital, from March 2007 to march 2008, with a detailed interview, complete otolaryngological examination, the Portuguese version of the Tinnitus Handicap Inventory and pure tone audiometry.

**Results:**

Age varied from 24 to 83 (mean=59); the mean THI value was 39 (females: 36; males: 44). THI grades were: slight: 32.3%; mild: 19.1%; moderate: 20.6%; severe: 13.2% and catastrophic: 14.7%. No significant correlation was found between gender (p=0.30), age (p=0.77) hearing loss (p>0.05 for all averages analyzed) and tinnitus severity.

**Conclusion:**

Gender, age and hearing loss do not influence tinnitus annoyance, using the THI.

## INTRODUCTION

Tinnitus is defined as the perception of sound in the ears or head with no external source of sound. It affects about 15% of the world population;^1^ this prevalence increases to 33% in individuals aged over 60 years.^2^

About 20% of patients with tinnitus find the symptoms difficult to bear, which significantly affects their quality of life. Several studies have attempted to investigate the causative factors of tinnitus.^3^, ^4^, ^5^, ^6^, ^7^, ^8^, ^9^, ^10^, ^11^, ^12^, ^13^, ^14^ Some have shown that the limitations due to tinnitus depend on primary psychological factors, such as difficulties in dealing with the problem,^8,^^12,^^15^, ^16^, ^17^ altered humor (depression and anxiety), low concentration, irritability, loss of control,^18^ and a variety of psychiatric conditions and specific personality traits.^12,^^15^, ^16^, ^17^,^19^

Contrary to what was commonly thought, Jastreboff and Hazell^2^ demonstrated that there are no psychoacoustic differences of tinnitus (intensity, frequency and minimum suppression level) among patients with tinnitus that suffer and those that do not. Other authors, however, have shown a small correlation between the intensity of tinnitus and its effect on patients.^19^, ^20^, ^21^ Thus, in medical practice, it is important to differentiate the intensity of tinnitus and how annoying it is for patients, since these parameters appear to correlate poorly.^11^

The influence of hearing loss on the degree of suffering caused by tinnitus remains uncertain.^22^ Weisz^23^ showed that severe tinnitus was associated with hearing loss for high frequencies. McKinney et al.^24^ found that clinically important hearing loss in tinnitus patients were associated with anxiety and depression as a reaction to hearing loss, which could affect the impact of tinnitus. It is thus still uncertain whether hearing loss is only a trigger for the onset of tinnitus or if it also predicts its severity and handicap.^24^,^25^ Searchfield et al.^13^ showed that low frequency hearing loss was correlated with increased annoyance due to tinnitus as assessed in the Tinnitus Handicap Questionnaire (THQ); the highest scores, however, were given to questions on hearing, rather than the total THQ score. In this same study, the Tinnitus Severity Index (TSI) - a questionnaire assessing how bothersome tinnitus is - did not correlate with any audiometric findings. Such poor correlation suggests that tinnitus patients are heterogeneous, and that several factors affect the impact of this symptom on the quality of life.

The prevalence of hearing loss and tinnitus increases with age.^3^,^26^ However, Meric et al.^7^ applied different questionnaires for evaluating the impact of tinnitus on quality of life, and found no correlation between age, sex or duration of tinnitus with the annoyance it generated. Davis^27^ and Coelho et al. (2004)^1^ found that female patients gave significantly higher annoyance scores compared to males. On the other hand, Hiller and Goebel^11^ encountered a higher severity (intensity and annoyance) due to tinnitus in older male patients.

Because of the subjective nature of tinnitus, its diversity of causes, and the heterogeneity of patients, this symptom is a complex topic to study and understand. Given such controversies, the purpose of this paper was to assess the influence of sex, age and degree of hearing loss on the annoyance patients felt due to tinnitus.

## MATERIAL AND METHOD

The institutional review board of our institution approved this study design and the free informed consent form (number 097/07), which met all the requirements for clinical studies on human beings.

There were 68 patients seen consecutively at the tinnitus outpatient unit of our institution from March 2007 to March 2008.

Inclusion criteria were as follows:

1 Age from 18 to 85 years.
2 Presence of unilateral or bilateral tinnitus lasting over 3 months.
3 Ability to answer the proposed questions.
4 Pure tone audiometry showing sensorineural dysacusis of any degree.

An acute or chronic infection of the external or middle ear was an exclusion criterion.

All patients were evaluated with the same systematic protocol for tinnitus patients and sound intolerance developed by Sanchez. This protocol was the basis for selecting the epidemiological and clinical data and the features of tinnitus and correlated symptoms for this study.

Patients then filled in the Brazilian version of the Tinnitus Handicap Inventory (THI),28,29 a 25-item questionnaire answered with “yes” (4 points), “no” (0 points) or “sometimes” (2 points). The scores are added to yield a classification of the tinnitus handicap, from negligible (0 to 16), mild (18 to 36), moderate (38 to 56), severe (58 to 76) or catastrophic (78 to 100).

The next step was a full clinical otorhinolaryngological and head & neck examination followed by pure tone audiometry in an acoustically treated booth to test frequencies at 250, 500, 1000, 2000, 3000, 4000, 6000 and 8000 Hz (Amplifon AMPLAID 460 audiometer, with conventional Telephonics 296 D 100-1 earphones).

The statistical analysis was done based on sex, age, THI scores and pure tone thresholds, as follows:

• conventional tritone mean (mean tri) - arithmetic mean of pure tone thresholds at 500, 1000 and 2000 Hz
• quadritone mean (mean quadri) - arithmetic mean of pure tone thresholds at 500, 1000, 2000 and 3000 Hz
• mean of all tested frequencies (mean total) - arithmetic mean at 250, 500, 1000, 2000, 3000, 4000, 6000 and 8000 Hz
• tritone mean at higher frequencies (mean acute) - arithmetic mean at 4000, 6000 and 8000 Hz.

These means were ipsilateral to tinnitus in patients with unilateral tinnitus, and ipsilateral to the side with worst tinnitus in patients with asymmetric bilateral tinnitus. The side with the worst tone means was used in symmetrical cases.

The Statistical Analysis System (SAS) software, version 9.1, was used for statistical calculations. The statistical tests included Wilcoxon's rank sum test and the Kruskal-Wallis non-parametric test for differences between means to correlate sex and the THI. Pearson's coefficient yielded the p values associated with the test statistics for numerical correlations (age, different means calculated based on pure tone audiometry and the THI).

## RESULTS

There were 68 patients, 27 males (39.7%) and 41 females (60.3%).

Age ranged from 24 to 83 years (mean - 59 years). The mean age among females was 61 years and the mean age among males was 57 years. Table 1 shows the standard deviation and variation coefficients. There were no statistically significant differences between the mean ages of both sexes (p=0.30).

**Table 1:** Means, standard deviation, and variation coefficient of age in patients.

	General	Females	Males
Mean Age	59	61	57
Standard Deviation	12	11	13
Variation Coefficient	20	18	22

Table 2 shows the final mean of the THI results according to each sex and the standard deviation and variation coefficient. There were no statistically significant differences in the THI scores according to sex according to the Kruskal-Wallis test (p=0.30), shown on Table 2.

**Table 2:** Means, standard deviation and variation coefficient for THI values.

	Total	Females	Males
Mean THI	39	36	44
Standard deviation	28	27	31
Variation coefficient	73	70	69

The THI scores were: slight: 22 patients (32.3%); mild: 13 patients (19.1%); moderate: 14 patients (20.6%); severe: 9 patients (13.2%); and catastrophic: 10 patients (14.7%). Chart 1 shows the THI scores according to sex.

The Wilcoxon rank-sum test revealed no statistically significant correlation (p value 0.30) between sex and the annoyance of tinnitus.

Pearson's correlation coefficient revealed no statistically significant correlation (Pearson's coefficient −0.03 and p value 0.77); it was not possible to predict the severity of tinnitus based on the patient's age.

Chart 2 shows the THI scores in different age groups. There were 7 patients (10%) under age 45 years, 39 patients (57%) aged from 45 to 65 years, and 22 patients (32%) aged over 65 years.

**Graph 1:**
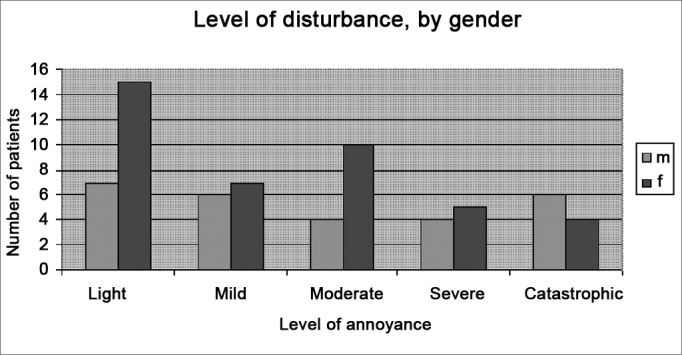
THI grading of the patients assessed at work, broken down by gender.

**Graph 2:**
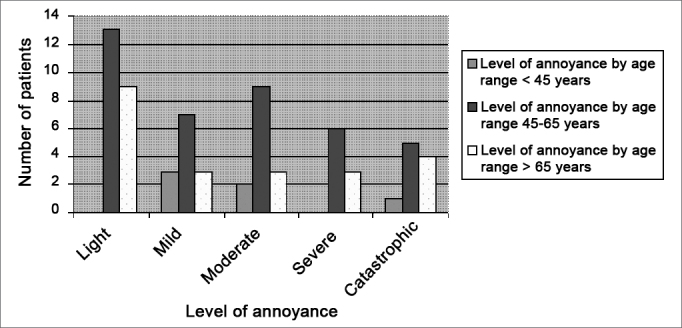
THI levels, broken down by age range.

The correlation of means in pure tone audiometry and the THI were:

a) Conventional tritone mean - mean tri (500, 1000, 2000Hz):
  There was no statistically significant correlation between the conventional tritone mean and the THI score (Pearson's correlation coefficient: 0.122 and p value: 0.32).

**Table 3:** p value of numerical THI correlations.

	Age	Mean Tri	Mean Quadri	Mean Total	Mean High-Frequency
THI	0,77	0,32	0,11	0,16	0,13

**Table 4:** Published papers correlating annoyance due to tinnitus with gender.

Author	Year	Assessment tool for annoyance due to tinnitus	Correlation with gender
Davis^34^	1983	Description mild, moderate, severe discomfort	Predominantly females
Meric et al.^7^	1998	THQ, TRQ, STSS	No correlation
Erlandsson and Holgers^9^	2001	TSQ	No correlation
Coelho et al.^1^	2004	EVA	Predominantly females
Hiller and Goebel^11^	2006	Mini-TQ	Predominantly males
b) Quadritone mean - mean quadri (500, 1000, 2000, 3000Hz):
  After obtaining the arithmetic means of these frequencies, the correlation revealed no statistically significant results (Pearson's correlation coefficient: 0.19 and p value: 0.11).
c) Mean of all frequencies - mean total (250, 500, 1000, 2000, 3000, 4000, 6000, 8000Hz):
  The correlation of means shown above with the THI was not statistically significant (Pearson's correlation coefficient: 0.17 and p value: 0.16).
d) Tritone mean of high frequencies (4000, 6000, 8000Hz):
  There was no statistically significant correlation (Pearson's correlation coefficient: 0.18 and p value: 0.13).

## DISCUSSION

Patients complaining of tinnitus may present varying degrees of annoyance with this symptom, with variable impact on the quality of life. Two important factors associated with tinnitus should be differentiated: the intensity of the tinnitus signal and the severity of this symptom or the annoyance that it causes to the lives of patients.

Studies have been controversial on the effect of gender on the prevalence of tinnitus. Although some have described a slightly higher prevalence in females,^30^,^31^ others have suggested that the prevalence is higher in males;^19^,^32^ these studies rarely have statistical significance. A possible explanation for a higher prevalence in males may be that men are more exposed to occupational noise.^33^ Women, on the other hand, generally have more time to seek medical care, which may explain a higher prevalence in females.1 Women also predominated in our study.

Based on the final THI score, we found no difference in the annoyance due to tinnitus among males and females. These findings concur with those of Erlandsson and Holgers^9^ even though these author applied another questionnaire, the Tinnitus Severity Questionnaire (TSQ) which assesses the severity of tinnitus, quality of life issues, concentration difficulties, discomfort in silence, and depressive reactions. Our findings also concur with those of Meric et al.^7^ who noted that sex, age or duration of tinnitus did not affect the degree of annoyance with the symptom; these authors applied three questionnaires (the THQ, the Tinnitus Reaction Questionnaire, and the Subjective Tinnitus Severity Scale). Coelho, Sanchez and Bento^1^ found that female patients gave significantly higher annoyance scores in a visual-analog scale compared to males. Although these results were different as applied to female patients, their assessment tools were not the same, which makes it difficult to compare results. Davis^34^ also found that females predominated among patients with severe bothersome tinnitus or with altered sleep, which disagrees with our results.

A possible indirect influence of sex is depression. Dobie and Sullivan^35^ estimated the prevalence of major depression in the adult population at 5%, which increases to 10% in patients receiving any type of medical care. These authors estimated that the incidence of major depression during life was double in females (20%) compared to males (10%). As the severity of tinnitus is strongly correlated with the presence of depression, it may be assumed that bothersome tinnitus occurs more often in females, as Davis ^34^ suggests, even though this was not evident in our study. Most studies have shown that the limitations (and even disability) generated by tinnitus depend to a large extent on primary psychological factors, such as difficulty in dealing with this problem.^8,^^12,^^15^, ^16^, ^17^ There is a strong correlation between the severity of tinnitus and altered humor (depression and anxiety), poor concentration, irritability, and loss of control.^15^ Most studies suggest fairly clearly a relation between the severity of annoyance due to tinnitus and the presence of psychiatric conditions and specific personality traits.^12,^^15^, ^16^, ^17^, ^19^,^19^

Hiller and Goebel (2006)^11^ found significant severity in terms of intensity and annoyance due to tinnitus in older male patients with binaural tinnitus and vertigo, hearing loss and hyperacusis, again contradicting our findings. The mini Tinnitus Questionnaire (mini-TQ) was used for assessing annoyance.

The prevalence of hearing loss and tinnitus increases with age, regardless of any history of occupation noise exposure. According to the National Hearing Study,^3^ there is a trend for increasing bothersome tinnitus with age.

We found no correlation between bothersome tinnitus and age in our sample. Chart 2 shows an apparent increase of the THI scores in the 45 to 65 age group; however, this is due only to a larger number of patients with this age in our study.

Our findings, as mentioned above, concur with those of Meric et al.^7^ in that no correlation was found between age and annoyance generated by tinnitus, based on the THQ, TRQ and STSS questionnaires. Hiller and Goebel^11^ on the other hand, found increased annoyance in older patients, which diverges from our findings.

Of note is the influence, attention and silence on the perception and annoyance due to tinnitus. Studies by Heller and Bergman^36^ and Knobel and Sanchez^37^ have suggested that tinnitus is a common subaudible phenomenon that may be perceived in silent ambiences or during heightened auditory perception.^37^ Thus, it may be inferred that older patients, having less work, remain longer in their homes, where silence and auditory attention could possibly be more relevant for increasing the perception of annoyance due to tinnitus. Brown^26^ found that subjects who do not work tend to present more tinnitus, but did not comment on this association. In Brazil there are many retired persons who continue to work on regular jobs. Surveys done by the Legislative Advisory Group of the House of Representatives^38^ and the Applied Economic Survey Institute (IPEA)^39^ have shown a high participation of retired elderly Brazilians in the labor market. Over half of elderly males and nearly one third of elderly females in the labor market had been retired, and that this tends to increase. In 2003, 46% of older men were working in regular jobs. The percentage of working women aged at least 60 years reached 19.6% in Brazil; only Nordic countries have higher percentages. This may explain why we found no correlation between age and bothersome tinnitus in our sample, since many elderly persons continue to work.

Tinnitus has been associated with almost all ear abnormalities, particularly with cochlear conditions.^40^ Hearing loss - especially severe loss - may be an added handicap to tinnitus, generating additional discomfort to patients; rather than affecting tinnitus directly, this adds to the general health problems that patients face.

We found no correlation between the severity of tinnitus and the degree of hearing loss. Holgers^19^ however, has shown a moderate association between the severity of tinnitus and audiometric parameters, such as the tritone mean.^19^

Axelsson and Ringdahl^30^ concluded that tinnitus is more common and severe in patients with hearing loss, which diverges from our findings.

Coles^3^ showed that the severity of tinnitus was correlated with auditory difficulty, finding mild hearing loss in patients with tinnitus that were mildly bothered and severe or profound hearing loss in patients with severely bothersome tinnitus, again diverging from our findings. This study also found that the chance of having moderately to severely bothersome tinnitus increased hand in hand with auditory thresholds at high frequencies.

Weisz^23^ showed that increased hearing loss at high frequencies were associated with a lower severity of tinnitus.

Holgers^8^ found that absence from work due to tinnitus were more frequent in patients with higher degrees of hearing loss. We may infer that these patients are more bothered with tinnitus, justifying their absence of work by associating annoyance with worse hearing.

Baskill and Coles^22^ have suggested that the influence of hearing loss on the severity of tinnitus remains uncertain; these authors found that auditory thresholds and bothersome tinnitus were poorly correlated, which is similar to our findings.

Savastano^14^ evaluated the relation between the THI score and the presence or absence of hearing loss in patients with tinnitus. THI results revealed that, in most cases, a slight or mild groups in the THI was attributed to individuals with hearing loss; normal hearing patients with tinnitus were more frequent in the moderate and catastrophic groups, which was statistically significant compared to the hearing loss group. Savastano^14^ used the same tool for evaluating bothersome tinnitus that we applied in our study (THI) and found that more severe hearing loss did not correlate with the severity of bothersome tinnitus, similar to our findings.

Sanchez et al.^41^ monitored tinnitus patients with normal pure tone audiometries and found that within a mean time of 3.5 years, 44.6% progressed with hearing loss, and that the majority of subjects in this group reported no change or improvement of tinnitus during this period. This finding may represent natural habituation with tinnitus in these patients.

Hallberg and Erlandsson^42^ found worse auditory thresholds in patients that reported more concentration difficulties and sleep disorders due to tinnitus, compared with patients with no complaints of tinnitus; these authors found that bothersome tinnitus was not more severe in patients with more severe hearing loss, again similar to our findings.

McKinney, Hazell and Graham^24^ found that more severe hearing loss in patients with tinnitus was associated with more frequent depression and anxiety, which could affect the impact of tinnitus on their quality of life. For many authors, it is not certain whether hearing loss is only a trigger for the onset of tinnitus or whether is also affects the severity and limitations of this symptom.^22,^^24,^^25^

Meric et al.^7^ found that tinnitus had more impact if there was associated hearing loss; patients without hearing loss appeared to have less bothersome tinnitus, which diverges from our results.

Searchfield et al.^13^ correlated the TSI and the THQ with hearing loss as assessed with pure tone audiometry, and found that hearing loss at low frequencies correlated with more severe bothersome tinnitus when evaluated by the THQ; significance was found only in the part of the questionnaire that contains questions on hearing, and not in the total score. The TSI did not correlate with any aspect of audiometry. The TSI is a shorter questionnaire that does not correlate with hearing loss; it only evaluated the degree of bothersome tinnitus. The correlation between hearing loss and the level of annoyance with tinnitus was poor in that study, which concurs with our findings.

We noted that findings in several studies vary widely, reflecting the idea that tinnitus patients are a heterogeneous group, and that many factors determine the impact of tinnitus on the lives of patients.

## CONCLUSION

According to the tool applied in this study, we concluded that the sex and age of patients, and the degree of hearing loss, have no influence on the annoyance generated by tinnitus in each patient.

## References

[1] Coelho CCB, Sanchez TG, Bento RF (2004). Características do zumbido em pacientes atendidos em serviço de referência. Arq Int Otorrinolaringol.

[2] Jastreboff PJ, Hazell JWP. (1993). A neurophysiological approach to tinnitus: clinical implications. Br J Audiol.

[3] Coles RRA. (1984). Epidemiology of tinnitus: prevalence. J Laryngol Otol.

[4] Erlandsson SI, Hallberg LRM, Axelsson A. (1992). Psychological and audiological correlates of perceived tinnitus severity. Audiology.

[5] Sullivan M, Katon W, Russo J, Dobie R, Sakai C. (1993). A randomized trial of nortriptylin for severe chronic tinnitus. Effects on depression, disability and tinnitus symptoms. Arch Intern Med..

[6] Russo J, Katon W, Sullivan M, Clark M, Buchwald D. (1994). Severity of somatization and its relationship to psychiatric disorders and personality. Psychosomatics.

[7] Méric C, Gartner M, Collet L, Chéry-Croze S. (1998). Psychopathological profile of tinnitus sufferers: evidence concerning the relationship between tinnitus features and impact on life. Audiol Neurootol.

[8] Holgers KM, Erlandsson SI, Barreñas ML (2000). Predictive factors for the severity of tinnitus. Audiology.

[9] Erlandsson SI, Holgers KM (2001). The impact of perceived tinnitus severity on health-related quality of life with aspects of gender. Noise Health.

[10] Jastreboff PJ, Hazell JWP. (2004). Tinnitus Retraining Therapy: Implementing the Neurophysiological Model.

[11] Hiller W, Goebel G. (2006). Factors influencing tinnitus loudness and annoyance. Arch Otolaryngol Head Neck Surg..

[12] Langguth B, Kleinjung T, Fischer G, Hajak P, Eichhammer P, Sand PG (2007). Tinnitus severity, depression and the big five personality traits. Prog Brain Res..

[13] Searchfield GD, Jerram C, Wise K, Raymond S. (2007). The impact of hearing loss on tinnitus severity. The Australian and New Zealand J Audiol.

[14] Savastano M. (2008). Tinnitus with and without hearing loss: are its characteristics different?. Eur Arch Otorhinolaryngol.

[15] Tyler RS, Baker LJ (1983). Difficulties experienced by tinnitus sufferers. J Speech Hear Disord.

[16] Unterrainer J, Greimel KV, Leibetseder M, Koler T. (2003). Experiencing tinnitus: which factors are important for perceived severity of the symptom?. Int Tinnitus J..

[17] McKenna L, Hallam RS, Hinchcliff R. (1991). The prevalence of psychological disturbance in neurotology patients. Clin Otolaryngol Allied Sci..

[18] Hallam RS, Jakes SC, Chambers C, Hinchcliff R. (1985). A comparison of different methods for assessing the intensity of tinnitus. Acta Otolaryngol.

[19] Holgers KM, Zoger S, Svedlund K. (2005). Predictive factors for development of severe tinnitus suffering further characterization. Int J Audiol.

[20] Kuk FK, Tyler RS, Russel D, Jordan H. (1990). The psychometric properties of a tinnitus handicap questionnaire. Ear Hear.

[21] Newman CW, Wharton JA, Shivapuja BG, Jacobson GT (1994). Relationship among psychoacoustic judgments, speech understanding ability and self-perceived handicap in tinnitus subjects. Audiology.

[22] Baskill JL, Coles RRA., Hazell J. (1999). Sixth International Tinnitus Seminar,.

[23] Weisz N, Voss S, Berg P, Elbert T. (2004). Abnormal auditory mismatch response in tinnitus sefferers with high-frequency hearing loss is associated with subjective distress level. BMC Neurosci.

[24] McKinney CJ, Hazell JWP, Graham RL (1999). An evaluation of the TRT method. Proceedings of the Sixth International Tinnitus Seminar.

[25] Davis A., Reich G.E., Vernon J.A. (1996).

[26] Brown SC (1990). GRI monograph series A.

[27] Davis AC, Lutman M.E., Haggard M.P. (1983). Hearing Science and Hearing Disorders.

[30] Axelsson A, Ringdahl A. (1989). Tinnitus: a study of its prevalence and characteristics. Br J Audiol.

[31] Sanchez TG, Campos CAH, Costa HOO (2003). Tratado de Otorrinolaringologia.

[32] Lockwood AH, Salvi RJ, Burkard RF (2002). Tinnitus. N Engl J Med..

[33] Stouffer JL, Tyler RS (1990). Characterization of tinnitus by tinnitus patients. J Speech Hear Dis.

[34] Davis AC, Lutman M.E., Haggard M.P (1983). Hearing Science and Hearing Disorders.

[35] Dobie RA, Sullivan MD, Vernon JA (1998). Tinnitus Treatment and Relief.

[36] Heller MM, Bergman M. (1953). Tinnitus aurium in normally hearing persons. Annual Otology.

[37] Knobel KA, Sanchez TG (2008). Influence of silence and attention on tinnitus perception. Otolaryngol Head Neck Surg..

[38] Furtado A. Na Consultoria Legislativa da Câmara dos Deputados. A participação do idoso no mercado de trabalho brasileiro. Dis-ponível em http://www2.camara.gov.br/publicaes/estnottec/tema8/2004_13576.pdf. Acessado em 15 de outubro de 2008.

[39] Camarano, AA. O idoso brasileiro e o Mercado de trabalho. Da diretoria de estudos sociais do Instituto de Pesquisas Econômicas Aplicadas. Disponível em http://www.ipea.gov.br/pub/td/2001/td_0830.pdf. Acessado em 15 de outubro de 2008.

[40] Eggermont JJ (2007). Pathophysiology of tinnitus. Prog Brain Res..

[41] Sanchez TG, Mak MP, Pedalini MEB (2005). Evolução do zumbido e da audição em pacientes com audiometria tonal normal. Arq Int Otorrinolaringol.

[42] Hallberg L.R.M, Erlandsson S.I. (1993). Tinnitus characteristics in tinnitus complainers and noncomplainers. Br J Audiol.

